# Effects of biotransport and hydro-meteorological conditions on transport of trace elements in the Scott River (Bellsund, Spitsbergen)

**DOI:** 10.7717/peerj.11477

**Published:** 2021-06-28

**Authors:** Sara Lehmann-Konera, Waldemar Kociuba, Stanisław Chmiel, Łukasz Franczak, Żaneta Polkowska

**Affiliations:** 1 Institute of Earth and Environmental Sciences, Faculty of Earth Sciences and Spatial Management, Maria Curie-Skłodowska University, Lublin, Poland; 2 Department of Analytical Chemistry, Faculty of Chemistry, Gdańsk University of Technology, Gdańsk, Poland

**Keywords:** Metals, Transboundary pollutants, Long-range atmospheric transport, Surface water, Arctic, Glaciated catchment

## Abstract

The shaping of surface water chemistry in the Svalbard Archipelago is strongly dependent on the geology of the catchment and the process of long-range transport of atmospheric pollutants (LRATP). It was found that the dissolved trace elements in the Scott River, which catchment is characterized by a decreasing degree of glaciation, were of the natural origin (i.a. weathering and dissolution of local geological substratum). The exception was Zn originated from LRATP. The paper describe the influence changes in hydro-meteorological conditions and the presence of a seabird colony on the variability in the transport of trace elements within the Scott River catchment. The work assesses long-time fluctuations in the concentration of twenty five trace elements (i.a. Al, Cr, Cu, Pb, Sr, and Zn) from eighty-four surface water samples and their relation to changes in water discharge (Q), precipitation (P), pH, and dissolved organic carbon (DOC) at two river sites (with one being under the influence of the biotransport factor). Based on the results of matrix correlation and cluster analysis it was found that the additional load of DOC from the nesting site of *Larus Argentatus* in the mouth section of the river drastically changed the hydro-geochemical cycle of Co, Ni, Zn, Ga, Sr, Rb, Ba and U (0.30 < r < 0.51). Furthermore, the results of cluster analysis confirmed that the bird colony’s nesting site was strongly responsible for the presence of U, Rb, Zn, Ni and marine-derived nutrients (e.g. Se and Li). The discharge of glacier meltwater and the alkaline character of water have a negative effect on the dissolution of Li and Mn (−0.31 < r < −0.51), but positively affect the level of Rb and U (r = 0.31 and 0.35, respectively) due to it being washing out a seabird nesting colony in the mouth section of the Scott River. It was observed that the event of rises in air temperature and rain, which results in increased water discharge, caused an intense transport of the trace elements load. Moreover, results of the precipitation sensitivity coefficient factor (CF) proved that precipitation effect the occurrence of Li, Sr and U in the Scott River.

## Introduction

Even though the Arctic environment is no longer considered to be pristine, the Svalbard Archipelago, due to its geographic location and specific atmospheric conditions, is an excellent place to investigate long-range transport of trace elements (Ruman et al., 2012; Kozak et al., 2013). The most effective trace element global transport media from areas of lower latitudes (i.a. Eurasia, North America) to the Arctic are atmosphere (Pacyna et al., 1985; Maenhaut et al., 1989; AMAP, 2005; Bazzano et al., 2016) and ocean currents (Bazzano et al., 2014; Ardini et al., 2016; Grotti et al., 2017; Zaborska, Beszczyńska-Möller & Włodarska-Kowalczuk, 2017). Meanwhile, also very interesting is the role of migrating seabirds considered to be a biotic medium for transporting nutrients and pollutants on a local scale from marine to terrestrial environments. Their nesting sites are called “hot spots” of pollutants in the polar region (Sagerup et al., 2009; Michelutti et al., 2010; Ziółek, Bartmiński & Stach, 2017). The natural sources of trace elements are geological weathering, volcanic eruptions, sea salts, biogenic sources, and soil derived dust. While anthropogenic sources are recognized to originate from: mining, smelting, industrial emissions and agricultural activities, combustion of fossil fuels (e.g. coal, gasoline or oil), non-ferrous metal production, and waste incineration (AMAP, 2005; Bazzano et al., 2014; Zaborska, Beszczyńska-Möller & Włodarska-Kowalczuk, 2017).

The presence of trace elements in surface water was studied in different components of the Svalbard Archipelago’s environment such as: glacier surface ice (Chmiel, Reszka & Rysiak, 2009; Lehmann et al., 2016), snow deposited on glacier surface (Kozioł et al., 2021), supraglacial streams (Jóźwiak, 2007), glacial rivers (Chmiel, Reszka & Rysiak, 2009; Zhang et al., 2015; Kozak et al., 2016; Kosek et al., 2019; Stachnik et al., 2019), creeks of glacier-free catchments (Chmiel, Reszka & Rysiak, 2009; Kozak et al., 2015; Szumińska et al., 2018) and lakes (Chmiel, Reszka & Rysiak, 2009; Szumińska et al., 2018; Kosek et al., 2019). The glacier retreat and thawing of permafrost accelerated rapidly in the 21^st^ century when the mean air temperature in High Arctic started to increase twice as fast as in any other place on Earth (Vaughan et al., 2013; Szumińska et al., 2018; Amélineau et al., 2019). Glaciers which have been until now receivers of transboundary pollutants from urbanized as well as industrialized areas of Eurasia and North America have started to be regarded as a secondary source of trace elements in the Arctic (Vaughan et al., 2013; Bazzano et al., 2014; Ardini et al., 2016; Grotti et al., 2017; Zaborska, Beszczyńska-Möller & Włodarska-Kowalczuk, 2017). It is expected that accelerated melting of glaciers will favour the release of more concentrated pulse of contaminants stored in glacier ice for years (AMAP, 2011). Studies regarding the presence of trace elements in the glacial meltwater have been mostly concerned with single metals such as Fe (Zhang et al., 2015) and Al (Stachnik et al., 2019). There have also been studies on a selected heavy metals such as Cd, Cu, Mn, Pb, and Zn (Drbal, Elster & Komárek, 1992; Chmiel, Reszka & Rysiak, 2009) as well as studies that have presented results for more than twenty trace elements in a small number of samples (Kozak et al., 2016; Lehmann et al., 2016; Kosek et al., 2019). The results for long-term monitoring of trace elements in relation to meteorological conditions was only conducted in a non-glaciated catchment of Fuglebekken (Hornsund) and did not consider the hydrological conditions (Kozak et al., 2015). Therefore, there is a knowledge gap about relationship between hydrological conditions and concentrations of trace elements in the glaciated catchments of the Svalbard.

The main objectives of this paper are to fill a knowledge gap about the impact of hydro-meteorological conditions (water discharge (Q), precipitation (P)) on trace elements transport and to evaluate the influence of the nesting site of the herring gull (*Larus Argentatus*) on trace elements composition in the Svalbard glacial river. Here we present results of concentrations and loads of twenty five trace elements (Ag, Al, As, Ba, Be, Cd, Co, Cr, Cs, Cu, Ga, Hg, La, Li, Mn, Ni, Pb, Rb, Sr, Th, Tl, U, V, Zn) in eighty-four surface water samples collected from the Scott River and assess the relations between their concentrations and hydro-meteorological conditions as well as water pH and dissolved organic carbon (DOC).

## Materials and Methods

### Study area

**Figure 1:**
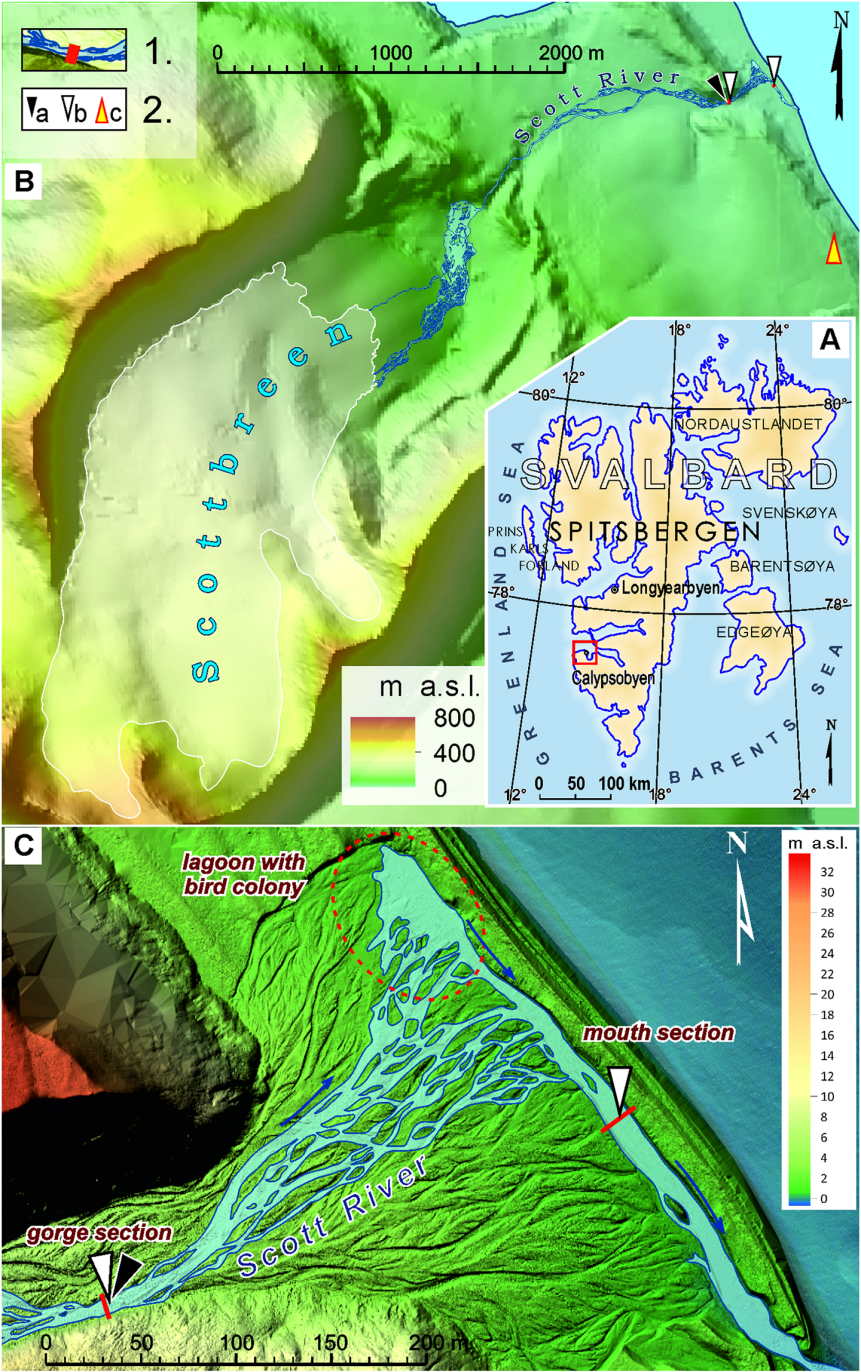
Study area. (A) Location of the study area in the Svalbard Archipelago. (B) Sampling and data collection sites in the area of the Scott River catchment (glacier-free part of valley with a variable channel pattern of the Scott River: 1. cross-section location, 2. samples and data collection sites (a. hydrological measurements, b. freshwater samples, c. meteorological measurements)). (C) Location of the ‘gorge’ and ‘mouth’ cross-sections in lower section of the valley. The red (dashed line) oval marks the periodic flow-through nesting site (coastal lake).

The Scott River which is located in the NW part of the Wedel-Jarlsberg Land (SW Svalbard) (Figs. 1A, 1B) drains a small glacial catchment area of 10 km^2^. The catchment area is approximately 40% occupied by the small Scottbreen valley glacier (Fig. 1B). The glacier, which is in a state of strong retreat (of rate about 20 m/y), delivers approximately 90% of water to the proglacial Scott River that runs into the Recherche Fjord (E part of the Bellsund). The main outflow of sub- and supraglacial streams is located in the SW part of the terminus at an elevation of about 90 m above sea level (a.s.l.).

The catchment area is varied, both in terms of surface sediments and landforms (Kociuba, 2017a). In the upper, glacierized section, there are partly metamorphosed upper and lower diamictites of the Kapp Lyell formation. Within the non-glacier covered part of the catchment area, these diamicitites are bordered (in SE) by a series of green and black shales and phylites, weathered sandstones, and conglomerates of the Bergskardet Formation. The central and lower parts of the catchment area are located within the Calypsostranda tectonic ditch which is filled with Paleogene deposits covered by Quaternary sea gravels. At the bottom of the non-glacier covered part of the Scott River valley there are Quaternary glacial, fluvioglacial, and fluvial deposits (Pękala, 2004; Harasimiuk & Gajek, 2013).

In geomorphological terms, the non-glacier covered valley floor can be divided into three sections which differ in terms of both spatial parameters and the predominant form of landforms and the geomorphic processes shaping them (Kociuba, Kubisz & Zagórski, 2014). The upper part of the valley floor is a wide (up to 600 m) inner marginal (Kociuba, Janicki & Dyer, 2019) an outwash fan, and enclosed by the ridge of the terminal moraine from NE side. The Scott River goes from the main outflow in the SW part of the glacier terminus gradually widening as a braided river up to the flow-through lake which forms yearly at the foot of the moraine ridge (Kociuba, 2017b) (Fig. 1B). From here, the waters of the Scott River overflow to the central section through a narrow (from a few to 50 m wide) rock throat (a gorge through the ridge of the terminal moraine). The central and lower part of the valley is indented into the raised marine terraces of ‘Calypsostranda’. In this part, the Scott River receives small tributaries, the largest of which is Reindeer Creek on its right-bank. The boundary between the central and lower part of the valley floor is formed by the narrow ‘lower’ gorge, cutting through the youngest and lowest raised marine terrace (25–30 m a.s.l.). On the slopes of the ‘lower’ gorge section, the outcrops of tertiary sandstones with hard coal inserts, sandstones, slates, and silts are visible. At the bottom of the gorge section, which locally narrows down from 150 to 50 m, the braided channels of the Scott River go into a single concentrated current, in order to disperse again below the gorge, on the surface of the alluvial fan. At the border of the alluvial fan’s base, the channels converge again and direct the outflow along the bank rampart to the SE by dissecting it in the S part of the fan and discharging into the fjord (Kociuba & Janicki, 2018). In the sections where the channels converge into one current, hydrometric measurement profiles and water gauges have been established (Fig. 1C).

### Sampling and data collection

All the daily freshwater sampling in the gorge (SRG) and the mouth (SRM) sections of the glacier-fed Scott River was conducted between July 13^th^ and August 22^nd^ of 2012 (Figs. 1B, 1C). The distance between two sites was approximately 200 m apart (Kociuba, 2017a, 2017b). The samples of surface water were manually collected into chemically clean 0.5 L plastic bottles made of high density polyethylene (HDPE) by personnel equipped with polyethylene gloves. In order to prevent the loss of analytes by their adsorption on the bottle’s wall, sample containers were washed with alkaline cleaning powder prior to the sampling. Subsequently, the bottles were cleaned a total of four times with ultrapure water (Mili-Q^®^ Ultrapure Water Purification Systems, Millipore^®^ production) by their week-long soaking, then emptying the water out of the bottles, and triple-rinsing them with the sample before its collection. To avoid the inflow of contaminants, the bottleneck was directed towards the water’s current. The loss of analytes to headspace was excluded by collecting the samples without any bubbles of air. Representativeness and composition stability of the freshwater samples were provided by their proper collection and cold storage (at approximately +4 °C) without access of light. In order to mitigate a possible impact of the sampling containers, a blank sample was used as a control.

Hydrological and meteorological measurements were performed at the location presented in Figs. 1B, 1C and collected with the use of methods described in Supplemental Material 1. In this study, hydro-meteorological data of Q and P were used to analyse the impact of hydro-meteorological conditions on trace elements transport.

### Analytical methods

Inorganic analysis of freshwater samples was performed after their delivery to the laboratory within 4 months of collection. Prior to the analysis, the samples were filtered through 0.45 µm-filter. Metals and metalloids quantitative analyses in surface water samples include the determination of Ag, Al, As, Ba, Be, Cd, Co, Cr, Cs, Cu, Ga, Hg, La, Li, Mn, Ni, Pb, Rb, Se, Sr, Th, Tl, U, V and Zn using the method of ion-coupled plasma mass spectrometry (Thermo Scientific XSERIES 2 ICP-MS, Germany, Collision Cell Technology, Cool gas flow Ar: 12 l/min, Cell gas flow He/H: 5.5 ml/min ). The trace elements analyses had the measurement range (MR), the limit of detection (LOD) and the limit of quantitation (LOQ) at three different levels. The first group of trace elements (Ag, As, Be, Cd, Co, Cr, Cs, Cu, Ga, La, Mn, Ni, Pb, Rb, Se Th, Tl, U, V) was characterised by MR between 0.010–1,000 µg/L, LOD = 0.010 µg/L and LOQ = 0.030 µg/L. Only Rb was characterised by MR in the range of 0.050–1,000 µg/L and LOD = 0.050 µg/L and LOQ = 0.150 µg/L. Finally the Al, Ba, Hg, L, Sr and Zn had MR of 0.100–1,000 µg/L, LOD of 0.100 µg/L and LOQ of 0.300 µg/L. Limit of detection (LOD) was calculated based on the standard deviation of the response (s) and the slope of the calibration curve (b) according to the formula: LOD = 3.3 (s/b). Limit of quantitation (LOQ) was calculated based on the standard deviation of the response (s) and the slope of the calibration curve (b) according to the formula: LOQ = 10 (s/b). Details of the analytical procedures which were used to perform the physicochemical analysis (pH) as well as to detect the sum parameter DOC are available in Lehmann-Konera et al. (2019).

### Quality assurance/Quality control (QA/QC)

To ensure the high quality of the obtained analytical results, all data was subject to strict quality control procedures. Due to the various matrix compositions of freshwater samples in the environment, there is a necessity to validate the analytical procedures which are applied in the determination of individual components against certified reference materials (Standard Reference Material NIST 1643e Trace Elements in Water, and Reference Material EnviroMAT ES-L-2 CRM, ES-H-2 CRM SCP SCIENCE). Moreover, the apparatus calibration phase involved using reference materials by Inorganic Ventures Analityk- CCS-4, CCS-6, CCS-1, IV-ICPMS-71A.

### Methods for results analysis

Calculations of the loads of dissolved trace elements transported to the neighbouring fjord were performed according to hydrological-hydrochemical methods used in Lehmann-Konera et al. (2018, 2019). Namely, *Li = Ci/Qi*, where *Li* is the calculated load of the analyte, *Ci* is the concentration of analyte, and *Qi* is the water discharge on a given day. Data for water discharge measured in the gorge section of the Scott River was used both for the calculations of loads at the mouth and the gorge section. The calculations of the elements load in the mouth section were performed with the use of water discharge data available for the gorge section.

The Student’s *t*-tests, Pearson’s correlation coefficient and hierarchical cluster analysis were computed with the software package STATISTICA 13.3 (TIBCO). Correlation coefficients were considered to be statistically significant at a level of significance where *p* < 0.05. The significance of differences in the mean of the concentration and load of the trace elements between compared two sections of the Scott River was determined by the Student’s *t*-test for two dependent trials. The cluster analysis performed on standardised data was used to detect a pairwise relationship between trace elements and DOC and to determine the differentiated origin of metals and metalloids in the river gorge and mouth sections. For the clustering authors used dendrogram analysis based on Ward’s method, with squared Euclidean distance. For the assessment of the possible impact of the hydro-meteorological and chemical indices on trace elements concentration in the gorge and mouth sections of the river, Pearson’s correlation coefficient (r) was calculated to detect pairwise relationships among meteorological conditions (P), hydrological conditions (Q), pH, DOC.

The sensitivity of the glacial river to the influence of precipitation and the degree of river water contamination in both river sections was calculated following the precipitation sensitivity coefficient factor method and contamination degree formula used by Kozak et al. (2016). Namely, *CF = C*_*0–1*_*/C*_*precipitation*_, where *CF* is the calculated contamination factor by the trace element, *C*_*0–1*_ is the arithmetic mean of analyte concentration in river water samples, while *C*_*precipitation*_ is the arythmetic mean of analyte concentration in precipitation. Meanwhile, Cdeg is the sum of CF calculated for the analysed trace elements.

## Results

### Inorganic chemistry features

**Figure 2:**
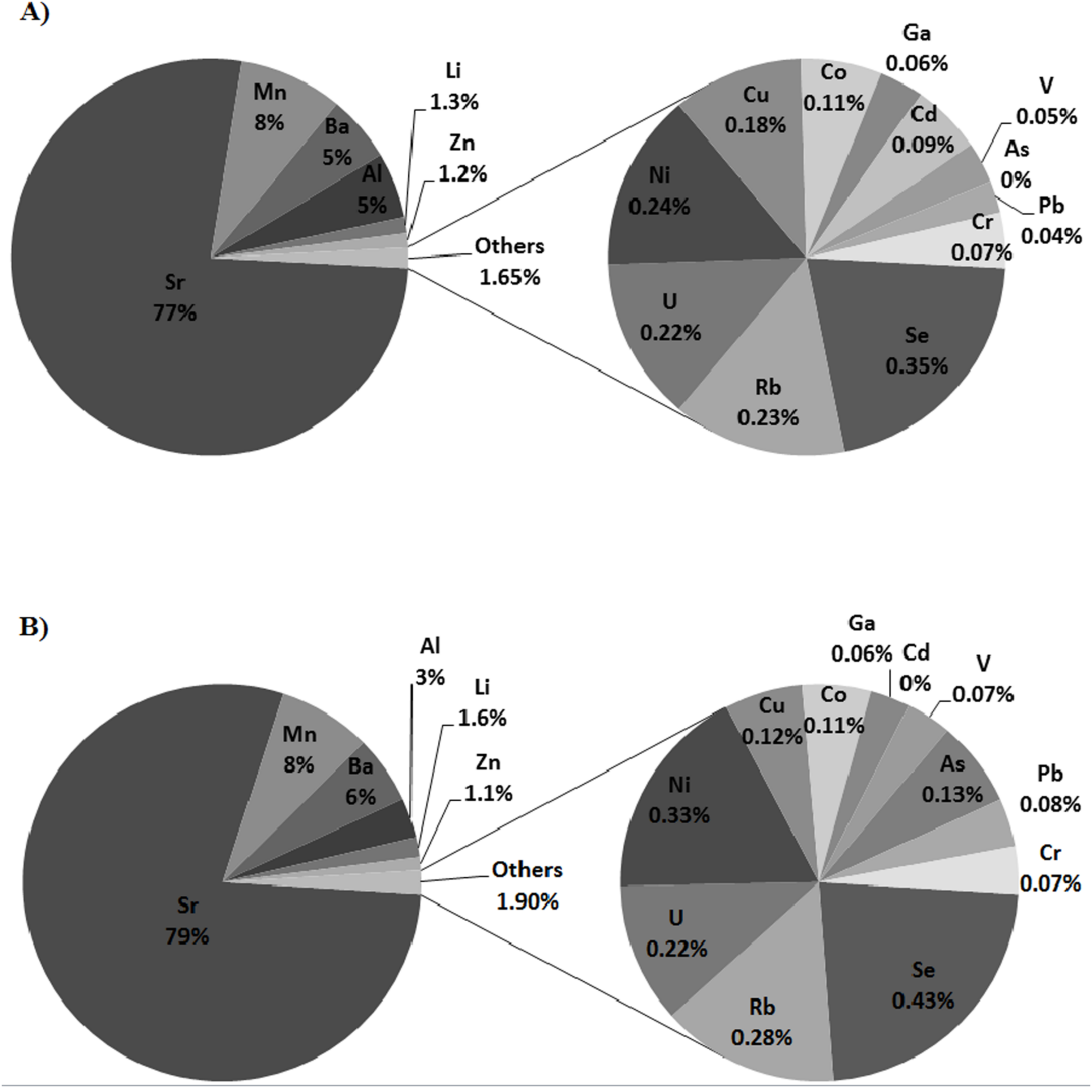
Percentage contribution in the mean value of trace elements in the freshwater samples collected in: (A) the gorge (SRG) and (B) mouth (SRM) sections of the Scott River.

The values of concentration and the load of determined dissolved trace elements were both presented in Table 1 and Supplemental File 2 and 3. Caesium (Cs), mercury (Hg) and lanthanum (La) were below the limit of detection (<LOD) in the freshwater samples collected from the gorge and the mouth sections of the Scott River. While silver (Ag) and cadmium (Cd) were also not detected in the mouth section. About 90 % of the results for Ag, beryllium (Be), thorium (Th) and thallium (Tl) in the river’s gorge were <LOD, and the same was true for the results of Be, Th and Tl in the mouth section. For this reason, these trace elements will be omitted in the Figs. 2A, 2B.

**Table 1:** Data of mean values of determined trace elements, pH and DOC in freshwater samples collected from the studied catchment and measured water discharge in the Scott River.

Analytes	*N*		Concentration (µg/L) Mean ± SD (median)		Load (µg/s) Mean ± SD (median)		Statistical difference of means (t)	
	Gorge	Mouth	Gorge	Mouth	Gorge	Mouth	Concentrations	Loads
Ag	1	0	n.d.	<LOD	n.d	n.d.	n.d.	n.d.
Al	42	42	1.62 ± 1.47 (1.18)	0.897 ± 1.12 (0.429)	1 467 ± 1 894 (887)	869 ± 1.312 (421)	3.24*	3.26*
As	0	38	<LOD	0.037 ± 0.025 (0.032)	n.d.	27.9 ± 15.7 (23.6)	n.d.	n.d.
Ba	42	42	1.65 ± 0.506 (1.66)	1.53 ± 0.454 (1.60)	1 446 ± 797 (1 311)	1 340 ± 765 (1 236)	1.36	0.93
Be	3	1	0.015 ± 0.002 (0.015)	n.d.	14.3 ± 3.60 (12.8)	n.d.	n.d.	n.d.
Cd	16	0	0.029 ± 0.022 (0.020)	<LOD	20.4 ± 19.9 (14.8)	n.d.	n.d.	n.d.
Co	42	42	0.033 ± 0.011 (0.032)	0.029 ± 0.011 (0.027)	29.4 ± 16.3 (26.3)	25.9 ± 16.6 (21.0)	1.84	1.49
Cr	10	14	0.010 ± 0.011 (0.010)	0.011 ± 0.018 (0.010)	24.1 ± 17.0 (19.1)	20.7 ± 11.3 (15.8)	−0.77	−0.32
Cs	0	0	<LOD	<LOD	n.d.	n.d.	n.d.	n.d.
Cu	33	42	0.054 ± 0.081 (0.026)	0.033 ± 0.020 (0.027)	45.9 ± 85.7 (20.7)	27.5 ± 16.2 (21.7)	0.92	0.82
Ga	41	42	0.018 ± 0.004 (0.018)	0.017 ± 0.004 (0.017)	16.6 ± 9.65 (14.9)	15.5 ± 8.89 (14.0)	1.49	1.05
Hg	0	0	<LOD	<LOD	n.d	n.d	n.d	n.d
La	0	0	<LOD	<LOD	n.d.	n.d.	n.d.	n.d.
Li	42	42	0.399 ± 0.082 (0.405)	0.444 ± 0.100 (0.444)	344 ± 149 (326)	383 ± 173 (367)	−3.68*	−2.69*
Mn	42	42	2.58 ± 3.74 (0.758)	2.08 ± 2.61 (0.945)	1 746 ± 2 608 (576)	1 622 ± 2 436 (543)	0.73	0.25
Ni	35	42	0.073 ± 0.077 (0.041)	0.092 ± 0.075 (0.079)	62.8 ± 75.7 (40.4)	80.9 ± 73.7 (60.9)	−1.85	−1.70
Pb	9	8	0.013 ± 0.003 (0.011)	0.021 ± 0.014 (0.017)	8.26 ± 4.94 (5.95)	22.7 ± 26.4 (11.3)	0.91	−1.19
Rb	42	42	0.072 ± 0.029 (0.068)	0.076 ± 0.018 (0.071)	65.8 ± 44.9 (52.2)	68.8 ± 40.2 (59.0)	−0.88	−0.60
Se	42	42	0.106 ± 0.036 (0.100)	0.119 ± 0.035 (0.122)	94.8 ± 59.3 (80.3)	105 ± 60.3 (78.6)	−2.10*	−1.85
Sr	42	42	23.4 ± 5.78 (23.3)	21.7 ± 5.42 (21.6)	20 664 ± 11 048 (19 378)	19 307 ± 10 863 (17 215)	1.76	1.10
Th	1	1	n.d.	n.d.	n.d.	n.d.	n.d.	n.d.
Tl	1	1	n.d.	n.d.	n.d.	n.d.	n.d.	n.d.
U	42	42	0.067 ± 0.045 (0.057)	0.059 ± 0.031 (0.050)	59.7 ± 48.8 (37.7)	53.8 ± 47.2 (33.6)	1.04	0.82
V	28	16	0.017 ± 0.012 (0.013)	0.011 ± 0.007 (0.010)	15.1 ± 11.7 (11.6)	17.2 ± 14.2 (13.7)	0.88	1.16
Zn	32	17	0.367 ± 0.327 (0.279)	0.133 ± 0.187 (0.100)	318 ± 300 (213)	255 ± 242 (116)	2.60*	2.32*
**Sum of metals**			30.4 ± 7.9 (29.6)	27.2 ± 6.6 (27.5)	26 291 ± 12 651 (24 072)	24 046 ± 12 740 (21 002)	2.19*	1.47
Chemical parameters**								
pH (-)			8.22 ± 0.262 (8.27)	8.21 ± 0.292 (8.19)	n.d.	n.d.	0.737	n.d.
DOC (mgC/L)			0.089 ± 0.054 (0.079)	0.098 ± 0.069 (0.081)	96.0 ± 85.4 (65.8)	99.6 ± 108 (68.8)	−1.43	−1.01
Hydrological parameter**			**Mean ± SD (median)**				n.d.	n.d.
Q (m^3^ s^−1^)			0.889 ± 0.404 (0.859)				n.d.	n.d.

**Notes:**

* Significant difference of means *p* < 0.05.

** Data after Lehmann-Konera et al., 2019.

*N*, number of samples with results >LOD used for loads calculation; n.d., not determined; S.D., standard deviation; t, student’s t-test.

A significant differences were observed between the concentrations of Al, Li, Se, and Zn in the samples collected from the gorge and mouth sections of the Scott River. While, a significant differences between loads were also noted for Al, Li, and Zn. As shown in Figs. 2A, 2B marked differences also occurred between the concentrations of arsenic and cadmium in these two sites.

Figure 2 shows a profile of the percentage contribution of trace elements in the gorge (SRG) and the mouth (SRM) section of the river. At both these sites, there prevailed a higher concentration of Sr, Mn, Ba and Al than of any other trace elements. Marked differences may be observed in the percentage contributions of Al, Li, Zn, Se, Ni, Cd and As.

**Figure 3:**
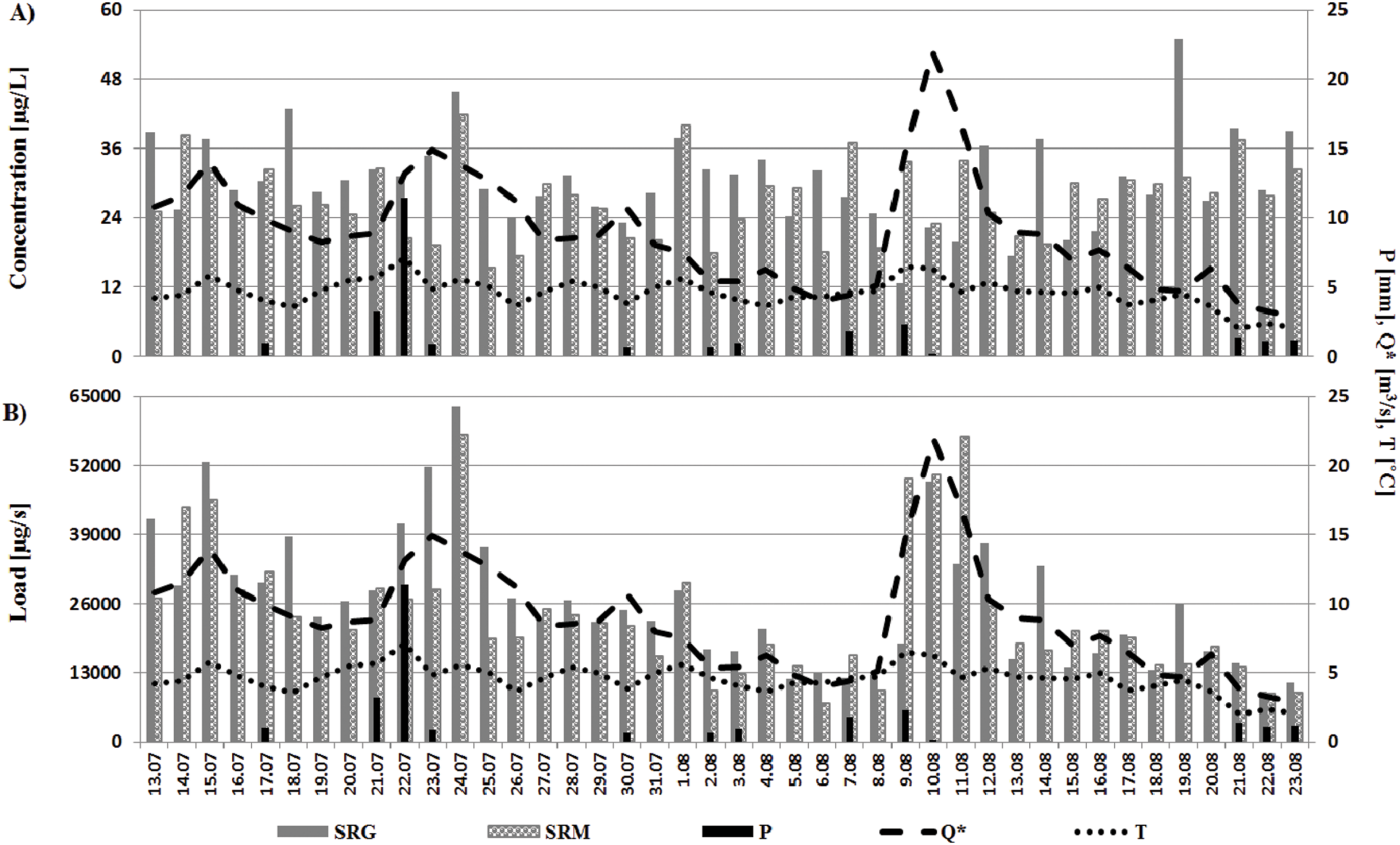
Variability of trace element concentrations (A) and loads (B) in the gorge and mouth sections of the river in relation to changes in hydro-meteorological conditions. Values of Q were multiplied by ten for better visualisation of the data.

The results of the metals and the metalloids analyses were studied in terms of the relations to the aforementioned hydro-chemical indices (Q, pH, DOC) which are presented in Lehmann-Konera et al. (Lehmann-Konera et al., 2019). During the measurement period, episodes of increased glacial river discharge were observed reaching their maximums on July 15^th^ and 23^rd^, and August 10^th^ (Lehmann-Konera et al., 2019). Figs. 3A, 3B demonstrates the influence of hydro-meteorological conditions on the changes in the concentration and load of trace elements in the measurement period.

The first episode of increased mean daily water discharge on July 15^th^ (1.40 m^3^/s) was the result of a rapid rise in mean air temperature to 5.79 °C and was accompanied by a higher concentration and load of trace elements in the gorge (37.5 µg/L, 52,557 µg/s) than in the mouth (32.4 µg/L, 45,429 µg/s) section of the river (Figs. 3A, 3B). While, the second one on July 23^rd^ (1.49 m^3^/s) was the result of both a heavy rain event (11.3 mm) and a rapid rise in mean air temperature to 7.09 °C on July 22^nd^ (Lehmann-Konera et al., 2019). On 23^rd^ of July there was observed higher concentration and loads of trace elements in the gorge (34.7 µg/L, 51,739 µg/s) than in the mouth (19.2 µg/L, 28,633 µg/s) of the Scott River. Continuous increases in river discharge noted on July 24^th^ (1.38 m^3^/s) and 25^th^ (1.27 m^3^/s) were the result of a re-increase of mean air temperature to 5.56 °C and 5.04, respectively. One of the highest values of trace element concentration (>40 µg/L) and the highest loads (>57,000µg/s) during the whole measurement period was observed in both sections on July 24^th^ (Figs. 3A, 3B). An increase of mean water discharge on August 9^th^ (1.47m^3^/s) was the result of lower than before precipitation event (2.28 mm) and a rapid rise in mean air temperature to 6.47 °C. The water discharge reached maximum on August 10^th^ and was accompanied by precipitation event (0.17 mm) and mean air temperature (6.29 °C). An increase in water discharge was sustained to August 11^th^ (1.70m^3^/s), even though the mean air temperature decrease to 4.65 °C (Lehmann-Konera et al., 2019).

During the episode of increased river discharge (between August 9^th^ to 11^th^), the transport of trace elements in the gorge section was mostly characterised by a lower concentration and load of trace elements but for a single-day increase in their transport (to 22.3 µg/L and 48,898 µg/s) which was a response to a maximum water discharge that occurred on August 10^th^. At the same time, the transport at the river’s mouth section was mainly characterised by a higher load of trace elements (between 49,412–57,465 µg/s) than the one in the gorge even though there was also a rapid decrease in the trace elements concentration (to 22.9 µg/L) in response to the said maximum water discharge on the 10th of August. Despite the highest concentration of trace elements being recorded on August 19^th^ their highest loads were observed in the gorge and mouth sections of the river on July 24^th^ (62,995 µg/s and 57,817 µg/s, respectively) (Figs. 3A, 3B).

### Cluster analyses (CA) and correlation matrix results

**Figure 4:**
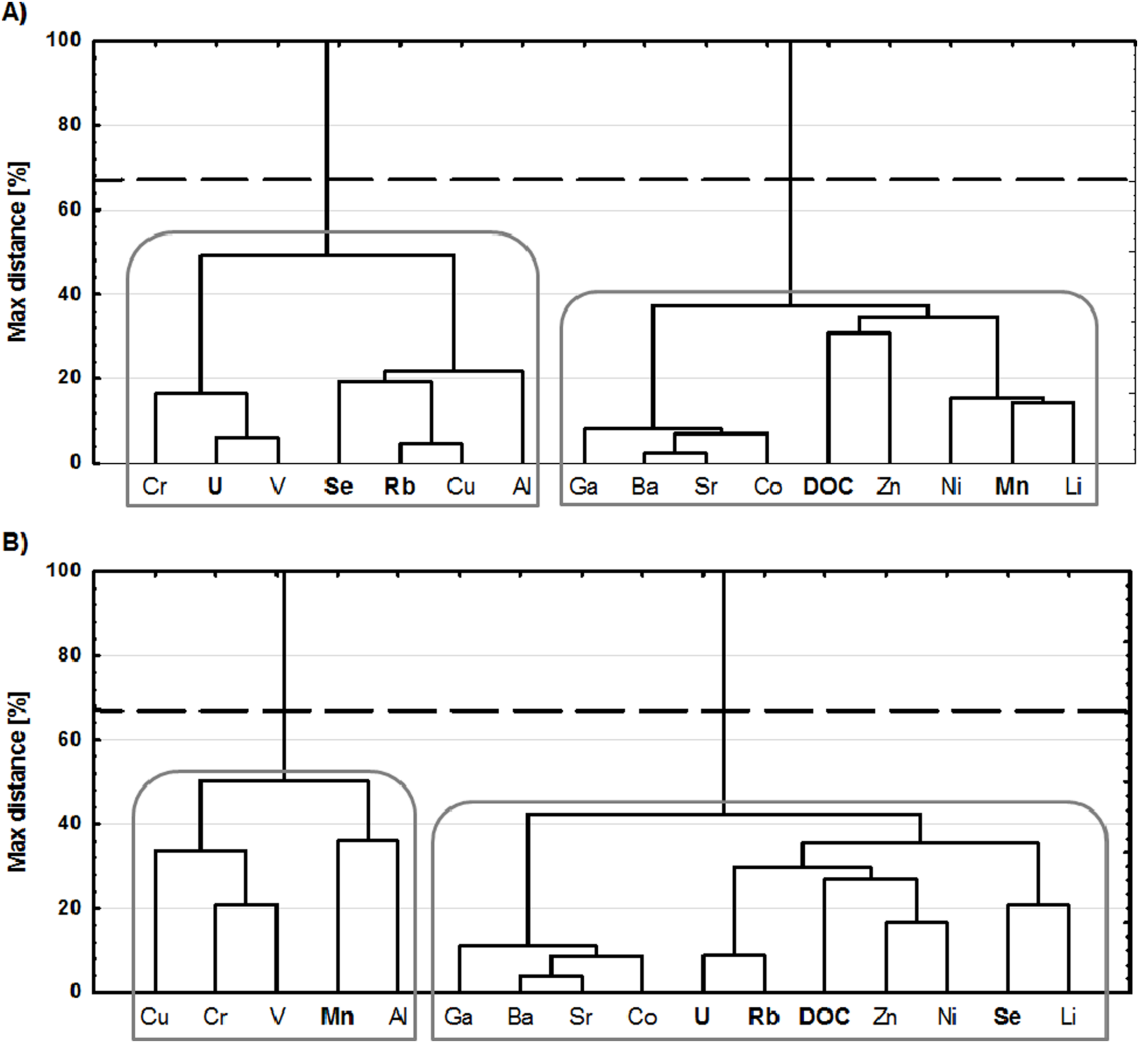
Hierarchical dendrograms resulting from cluster analysis of chemical indices in the gorge (A) and mouth sections (B) of the Scott River. A dashed line represents typical cutoff points (at a 67% relative distance level) for the most similar items. Major clusters were marked with a gray bold line.

Due to a large number of trace elements concentration results which were <LOD, only fifteen elements (Li, Al, V, Cr, Mn, Co, Ni, Cu, Zn, Ga, Se, Sr, Rb, Ba, U) allow performing the cluster analyses (Figs. 4A, 4B) and correlation matrix (Tables 2A, 2B). The cluster analysis (CA) has been applied to a set of sixteen variables (Figs. 4A, 4B) in order to group elements according to their maximum similarity. Two hierarchical dendrograms were based on the standardised data of summative parameter DOC and fifteen trace elements.

**Table 2:** Correlation matrix of the results of chemical analysis with hydro-meteorological parameters in the gorge and mouth sections.

	Li	Al	V	Cr	Mn	Co	Ni	Cu	Zn	Ga	Se	Sr	Rb	Ba	U	∑metals
**(A) SRG**																
**Q**	**−0.32**	0.05	−0.02	0.21	**−0.37**	−0.05	−0.05	−0.04	−0.04	0.13	0.04	−0.07	0.18	−0.12	−0.01	−0.23
**P**	0.09	0.10	−0.19	−0.11	−0.06	−0.03	0.05	−0.05	−0.05	−0.06	−0.14	−0.00	0.08	−0.01	−0.12	−0.18
**pH**	**−0.53**	0.14	0.15	−0.10	−0.30	0.00	−0.06	−0.02	0.12	−0.12	−0.11	−0.14	0.07	−0.16	0.20	−0.02
**DOC**	0.12	−0.23	−0.27	−0.10	−0.06	0.20	−0.03	−0.18	−0.08	0.05	−0.04	0.23	0.07	0.13	−0.10	0.10
**(B) SRM**																
**Q**	**−0.31**	0.16	0.15	0.15	−0.22	−0.03	−0.02	−0.26	−0.02	0.27	−0.05	0.01	0.21	−0.09	0.10	−0.01
**P**	−0.00	0.13	−0.10	−0.11	−0.03	−0.03	−0.08	−0.03	0.01	0.09	−0.11	−0.06	−0.03	−0.10	0.11	−0.04
**pH**	**−0.36**	0.14	0.16	−0.07	**−0.51**	0.18	−0.10	−0.20	0.13	0.17	0.01	−0.01	**0.31**	−0.02	**0.35**	−0.05
**DOC**	0.26	−0.22	0.04	0.05	−0.02	**0.51**	**0.39**	−0.24	**0.46**	**0.33**	0.07	**0.46**	**0.43**	**0.38**	**0.43**	**0.32**

**Notes:**

Statistical significances (*p* < 0.05) are marked in bold.

Cluster analysis results show, there were two major clusters of chemical indices formed in both sections of a river (C_1_SRG_, C_2_SRG_, C_1_SRM_ and C_2_SRM_) (Figs. 4A, 4B). Moreover, noticeable dissimilarities were noted in the individual trace elements associated with them. In the SRG section (Fig. 4A) C_1 and C_2 may be divided into: subcluster 1 (SC_1_SRG_): Cr, U and V; subcluster 2 (SC_2_SRG_): Se, Rb, Cu and Al; subcluster 3 (SC_3_SRG_): Ga, Ba, Sr and Co; subcluster 4 (SC_4_SRG_): DOC, Zn, Ni, Mn and Li. In the SRM section (Fig. 4B), C_1 represents the least numerous group of elements formed by SC_1_SRM_ (Cu, Cr and V) and SC_2_SRM_ (Mn and Al). While the second major cluster (C_2_SRM_) was the most diverse one. There could be observed two distinct groups of metals and metalloids within it: SC_3_SRM_ (Ga, Ba, Sr and Co) and SC_4_SRM_ (U, Rb, DOC, Zn, Ni, Se, Li). The SC_3_SRG_ and SC_3_SRM_ were formed by the same chemical indices, while in other subclusters in the gorge and mouth sections of a river could be observed a marked difference. The discrepancy in the arrangement of the chemical indices in major clusters was noticeable in the case of Mn, U, Se, and Rb. In the gorge section, Mn was associated with C_2, while in the mouth with C_1. Moreover, U, Se and Rb associated with the cluster C_1_SRG_ in the mouth section were observed in the C_2_SRM_.

The relationships between trace elements concentrations and the hydro-meteorological (Q, P) as well as the chemical parameters (pH, DOC) for the gorge and the mouth sections of the Scott River are presented in Tables 2A, 2B. The results and relationships between Q, P, pH, DOC were widely discussed in Lehmann-Konera et al. (2019). For the purpose of assessing the correlation degree between dissolved trace element concentrations and hydro-meteorological and chemical parameters the following scale, as in Stanisz (1998), was performed: +/− (r > 0.50)—correlation; +/− (r = 0.30–0.50)—suspected correlation; +/− (r < 0.30)—no correlation.

The correlation matrix results of the trace elements concentration and hydro-meteorological indices (Table 2) showed a significant negative correlations between concentration of dissolved Li and Q as well as water reaction (pH) at both river sites. A significant negative correlations were also noted between Mn and Q in the gorge, and between Mn and water pH in the mouth section. Whereas, the concentrations of Rb and U showed a significant positive correlation to water pH and DOC. The significant positive correlation also occurred between Co, Ni, Zn, Ga, Sr, Ba, ∑ of trace elements and DOC in the mouth section of the river. No correlations, however, was noted between the concentration of trace elements at both sites of the river and precipitation.

### Precipitation sensitivity coefficient index (CF)

Due to a limited number of trace elements which were determined in precipitation samples by an earlier study (Lehmann et al., 2016), the sensitivity of the glacial river to the influence of precipitation (CF) and the degree of river water contamination (Cdeg) in both river sections was performed only for selected metals.

Taking into consideration the results of the CF calculated for the gorge and mouth sections of the river (Table 3), it can be concluded that from among the elements which were chosen for calculation, U (CF > 6) had the greatest influence on the contamination of the study area, Li had a significant influence (3 ≤ CF < 6), and Sr had a moderate impact (1 ≤ CF < 3). Summing up the CF of the analysed trace elements allows us to assess the degree of contamination (Cdeg) which in both river sections was within the range of <8 Cdeg< 16. In turn, this indicates moderate contamination of the glacial river.

**Table 3:** Results of the contamination factor and the degree of contamination calculated for chosen trace elements in the gorge and mouth sections of the Scott River.

CF	C (P)	C (SRG)	C (SRM)	CF (SRG)	CF (SRM)
**Al**	2.59	1.62	0.897	0.625	0.346
**As**	0.042	–	0.037	–	0.809
**Cd**	0.423	0.029	–	0.069	–
**Co**	0.618	0.033	0.029	0.054	0.047
**Cr**	0.877	0.010	0.011	0.011	0.012
**Cu**	25.4	0.054	0.033	0.002	0.001
**Li**	0.096	0.399	0.444	**4.16**	**4.63**
**Mn**	5.11	2.58	2.08	0.505	0.408
**Ni**	1.36	0.073	0.092	0.054	0.068
**Pb**	0.773	0.013	0.021	0.017	0.027
**Sr**	10.1	23.4	21.7	**2.32**	**2.15**
**U**	0.010	0.067	0.059	**6.73**	**5.92**
**V**	0.230	0.017	0.011	0.074	0.047
			**Cdeg**	**14.6**	**14.5**

**Notes:**

Calculations were made with the use of the precipitation sensitivity coefficient factor method. The CF and Cdeg classification categories after Kozak et al. (2016): insignificant contamination (*CF* < 1 or *C*_deg_ < 8), moderate contamination (1 ≤ *CF* < 3 or 8 ≤ *C*_deg_ < 16), significant contamination (3 ≤ *CF* < 6 or 16 ≤ *C*_deg_ < 32), and heavy contamination (*CF* ≥ 6 or *C*_deg_ ≥ 32). Statistical significances (*p* < 0.05) are marked in bold.

## Discussion

The chemistry of surface water in Svalbard is mainly shaped by the processes of rock-water interaction and deposition of pollutants (wet and dry) (Dragon & Marciniak, 2010). The chemical feature of Svalbard’s surface water is also strongly shaped by the dissolution of calcium carbonate which is responsible for its alkaline character. The more acidic character of the water results in a higher concentration of metals (Kozak et al., 2015; Lehmann et al., 2016). Trace elements are supplied to the catchment area mainly by way of rock-weathering but they also originate from the atmosphere in form of dust, aerosols of both anthropogenic and natural origin, or as gaseous pollutants. They may be infused into surface water directly through wet and dry deposition but also indirectly through the rinsing off process in the glacier or tundra area (Jensen, 1999; Holt, 2000; Dragon & Marciniak, 2010). The lower degree of catchment glaciation results in a stronger effect on weathering of surrounding rocks and transport of material accumulated on the glacier surface (Ottesen, 2015).

### Natural and anthropogenic origin of trace elements

The trace element compositions at each of the two study sites on the Scott River differ significantly in the case of Al, Se and Zn. The first of the study’s sites (the gorge section) was the area of the catchment where trace element composition was the outcome of both natural (i.e. geological weathering, soil derived dust) and anthropogenic factors (wet and dry deposition of pollutants from local sources and long-range atmospheric transport). While the chemistry of the second point was additionally under a strong influence of the bird colony which uses this place as a nesting site during summer seasons. In 2012, there were not any volcanic eruptions recorded and so this natural source of trace elements was excluded from further investigation (Kozak et al., 2015). In gorge section, it can be noted that there was a lack of any relation between the concentration of DOC and trace elements concentration (Table 2A). While at the mouth, there was a significant positive correlation between DOC and Co, Ni, Zn, Ga, Sr, Rb, Ba and U (Table 2B). This indicates an anomaly caused by the presence of the bird colony at the mouth of the river.

The results of cluster analysis allow for distinguishing in both sections of a river two major sources of dissolved trace elements, namely local geological substratum (C_1) and deposition processes (C_2) (Figs. 4A, 4B). For the Scott River catchment is characteristic the mineralogical associations quartz-muscovite-chlorite-albite and calcite-dolomite. The bedrock of the Scott Glacier mainly consists of diamictite (quartzite and dolomite clasts) (Chmiel, Reszka & Rysiak, 2009; Lehmann et al., 2016). Unfortunately, the local rock composition of the area’s surroundings in the Scott River catchment is mostly limited to the concentration of Fe, Mn, Zn and Pb (Chmiel, Reszka & Rysiak, 2009). From the mineral composition of the suspension at the Scott River’s mouth in 2005, the following percentage contribution was assessed to exist: quartz (50%), dolomite (20%), calcite (11%), muscovite/serycyte (10%), plagioclase (albite) and chlorite (both at 4%), burnable organic matter (0.5%), Fe oxides and hydroxides (0.5%), and less than 0.5% of orthoclase. The aforementioned minerals as well as dust which derived from Bohlinrygen and Wijkanderberget rocks could be the natural source of Fe, Mn, Zn and even Pb in the Scott River. The presence of Fe, Zn, Sr, Al, Ba, Mn, Cu, Ni, Pb, Rb, Se and Cr on the Scott Glacier’s surface ice were the result of wet and dry deposition, both from natural (bedrock geology, soil and rock dust) and from anthropogenic (LRTAP and local pollution) sources. Glaciers are believed to be a source of Al that is released into water as a result of aluminosilicates being weathered under subglacial conditions which is enhanced by processes of hydrolysis, sulphide oxidation, and carbonation (Stachnik et al., 2019). The C_1_SRG_ and C_1_SRM_ were formed by the elements originating from bedrock and geological processes of the rock-weathering and dissolution. All of the elements in the C_1_SRG,SRM_ (Figs. 4A, 4B) were at a low concentration levels and occur in the overbank sediments of the studied area, what could indicate their natural origin from ore-bearing veins. Furthermore, the gorge section was located within the Calypsostranda tectonic ditch which is filled with Paleogene deposits, that have a distinct relation to occurrence of As and Cd (Ottesen, 2015). It explains the possible origin of Cd in samples collected in the SRG section (Fig. 2A).

The C_2_SRG, SRM_ presents the groups of elements derived to the catchment through deposition processes. Subcluster 3 in both sites of the river was represented by crustal elements (Ga, Ba, Sr and Co) (Maenhaut et al., 1989) that originated presumably from weathering of the surrounding rocks (Bohlinryggen massif and Wijkanderberget). DOC and Zn from SC_4_SRG_ (Fig. 4A) was most likely emitted to the atmosphere in the remote areas (Eurasia, North America) and transported to the Arctic in the process of the long-range atmospheric transport of pollutants. While, Ni, Mn and Li could be also related to LRATP (Maenhaut et al., 1989) as shown in Table 2 DOC have no correlations with these elements, and thus they were presumably supplied with marine aerosols.

Additional loads of DOC occurred in the mouth section of a river was related with the seabirds activity in this area (Lehmann-Konera et al., 2019). Migrating birds are one of the most important medium transporting pollutants and nutrients from marine to terrestrial environment in the High Arctic. The presence of Se, Cu, Cd and Zn was noted in the feathers, liver, kidney, muscle, gonads, and lungs of migrating birds which came from Norway (Wenzel & Gabrielsen, 1995). Moreover, Zn, Mn, Cu, Fe, Ni, Cr, Pb, Cd, Co were determined to be present in the feathers of dead *Larus Argentatus* collected at Adolfbukta and Bockfjorden locations (Svalbard) (Drbal, Elster & Komárek, 1992). Also, seabird colonies in the Dunderdalen were identified to be responsible for higher concentrations of Zn, Mn, Cu and Cd in the organic soils (Ziółek, Bartmiński & Stach, 2017).In the SC_4_SRM_ (Fig. 4B) U, Rb, DOC, Zn and Ni were presumably originated from the process of rinsing off organic matter and trace elements from the nesting site of the bird colony (Table 2B). Since in the Norwegian Arctic even 75% of atmospheric Se during summer is attributed to marine biogenic source (Maenhaut et al., 1989), Se and Li in SC_4_SRM_ were most likely related with deposition of marine aerosols. The seabird nesting colony (*Larus Argentatus*) strongly affects the hydro-geochemical cycles of trace elements in the mouth section of the Scott River as compared to said cycles observed in its gorge (Tables 2A, 2B, Figs. 4A, 4B). The reason for this effect was the additional load of organic matter that favors redox reactions, sorption, and peptisation which cause changes in metals concentration (Gong & Barrie, 2005). Thus the differentiated composition of major clusters in the gorge and mouth section of the Scott River reflect both the impact of seabirds colony on changes in the hydro-geochemical cycle of trace elements and also could indicate their role in the transport of Zn, Ni, U, Rb, Se, and Li from the marine environment.

The presented results indicate the *Larus Argentatus* nesting site to be the hot spot for trace elements such as Zn, Ni, U, Rb, Se, and Li. It must be noted that the role of seabirds as a means of biotransport for contaminants is not yet well-known. Also, the future environmental fate of trace elements which have accumulated in their nesting site should be the subject of further research with special attention being paid to the assessment of the balance between the inflow and outflow of pollutants within the terrestrial environment.

### Impact of hydro-meteorological conditions and water pH

During the summer season, the hydrology of Svalbard glacial rivers is more dependent on changes in air temperature than occurrence of precipitation (Nowak & Hodson, 2013). This was confirmed also by our research. The rapid rises in air temperature accompanied by precipitation events, in the measurement period of summer 2012, favors a rapid supply of freshwater from melting Scott Glacier (Franczak, Kociuba & Gajek, 2016). In consequence, it causes changes in the hydrological conditions of the Scott River and hence the release of intense pulses of organic carbon (Lehmann-Konera et al., 2019). Moreover, an increase of Q in thea river causes a decrease in the ionic strength of the solution which then hinders the formation of metal complexes in the water (Jensen, 1999; Holt, 2000). Meanwhile, an increase in Q was accompanied by an increased pH of the water (Lehmann-Konera et al., 2019). The Scott River was characterized by a neutral/alkaline pH which hindered metals from appearing in the ionic form, and this could be harmful to the environment due to these metals’ potential toxicity (Jensen, 1999; Holt, 2000). Consequently, it explains the significant negative correlation between Q, pH and trace elements (Li, Mn) in the gorge and mouth sections of the river (Table 2). It also clarifies the significant positive relationships between pH, Rb and U that were rinsed off the lagoon, which was a nesting site for a seabird colony. Even though results of the correlation matrix do not support the influence of Q on changes in the concentration of trace elements (Table 2), the events of increased discharge which were observed in the Figs. 3A, 3B. confirm that an intense burst of glacial freshwater was responsible for rinsing off trace elements of the Scott Glacier (mostly in July) and of the nesting site of *Larus Argentatus* (in early August). If climate warming should lead to more extreme character of weather events in the future, we may then observe a greater influence of catchment hydrology on trace elements transport.

Purifying rains are considered as one of the exchange route of pollutants between the atmosphere and the earth’s surface (Macdonald et al., 2000). Precipitation is a source of organic pollutants as well as trace elements (Kozak et al., 2015; Lehmann et al., 2016). In the summer season of 2012, precipitation pH ranged from 5.98 to 7.93 and was a source of Al, As, B, Cd, Co, Cr, Cs, Fe, V, La, Mn, Ni, Pb, Sr and U from LRATP (Lehmann et al., 2016). In the Revelva catchment characterized by an acidic/neutral pH (3.86-7.00), the acidic character of precipitation was responsible for increases in metal mobility in the river and resulted in their higher concentration (Kozak et al., 2015). Even though results of the matrix correlation show no significant relationships between precipitation and levels of trace elements in the Scott River (Tables 2A, 2B), the results of CF for Sr, Li and U (Table 3) may be seen to indicate that P plays an important role in their occurrence in the proglacial river. Meanwhile, the concentration of most of the elements which were used for calculating Cdeg was deemed to be on the level of “average pollution” (Table 3) and should be mostly treated as natural background.

## Conclusions

This study shows that the presence of trace elements in the Scott River water during the melt season of 2012 originated predominantly from natural sources. Trace elements were supplied to the surface water from processes of weathering and dissolution of local geological substratum (Al, Mn, Cd, Cr, Cu, Se, Rb, U, V) and deposition of mineral dust (Ga, Ba, Sr, Co). Next, the nesting site of the *Larus Argentatus* which was located in the mouth section of the river appeared to be another viable source of metals in the glaciated catchments which were derived from marine environment like U, Rb, Zn, Ni, Se, Li and probably As. This *Larus Argentatus* bird colony was responsible for the additional load of DOC in the river’s water which, in consequence, dramatically changes the hydro-geochemical cycles of the Co, Ni, Zn, Ga, Sr, Rb, Ba and U in the lower part of the river. Only the presence of DOC and Zn in the gorge section of the Scott River were related to the process of long-range atmospheric transport of pollutants from remote areas of Eurasia and North America to Svalbard.

The direct effect of hydro-meteorological conditions on the trace elements transport could only be observed during the rapid rises in air temperature accompanied by precipitation, which were responsible for the release of more intense pulses of trace elements from the Scott River catchment. Melting of surface ice intensified by rise in air temperature and precipitation, leads to rinsing off the trace elements from the Scott Glacier surface and nesting of seabirds colonies.

It proves that more frequent occurrences of heavy rain or further rises in the Arctic’s air temperature may very well lead to an increased transport of trace elements from the terrestrial environment to marine ecosystems.

## Supplemental Information

10.7717/peerj.11477/supp-1 Supplemental Information 1 Methodology of hydro-meteorological measurements.

10.7717/peerj.11477/supp-2 Supplemental Information 2 Raw data of trace elements concentration.

10.7717/peerj.11477/supp-3 Supplemental Information 3 Data of minimum and maximum values of determined trace elements in freshwater samples collected from the studied catchment. n.d.- not determined, (N)-number of samples with results >LOD used for loads calculation, * data after Lehmann-Konera et al., 2019.

## Additional Information and Declarations

### Data Availability

The following information was supplied regarding data availability: The data of trace elements values are available in the Supplemental File.

## References

[ref-1] AMAP (2005). AMAP assessment 2002: heavy metals in the Arctic.

[ref-2] AMAP (2011). Snow, water, ice and permafrost in the Arctic (SWIPA): climate change and the cryosphere.

[ref-3] Amélineau F, Grémillet D, Harding AMA, Wojciech W (2019). Arctic climate change and pollution impact little auk foraging and fitness across a decade. Scientific Reports.

[ref-4] Ardini F, Bazzano A, Rivaro P, Soggia F, Terol A, Grotti M (2016). Trace elements in marine particulate and surface sediments of Kongsfjorden, Svalbard Islands. Rendiconti Lincei.

[ref-5] Bazzano A, Cappelletti D, Udisti R, Grotti M (2016). Long-range transport of atmospheric lead reaching Ny-Ålesund: inter-annual and seasonal variations of potential source areas. Atmospheric Environment.

[ref-6] Bazzano A, Rivaro P, Soggia F, Ardini F, Grotti M (2014). Anthropogenic and natural sources of particulate trace elements in the coastal marine environment of Kongsfjorden, Svalbard. Marine Chemistry.

[ref-7] Chmiel S, Reszka M, Rysiak A (2009). Heavy metals and radioactivity in environmental samples of the Scott glacier region on Spitsbergen in summer 2005. Quaestiones Geographicae.

[ref-8] Dragon K, Marciniak M (2010). Chemical composition of groundwater and surface water in the Arctic environment (Petuniabukta region, central Spitsbergen). Journal of Hydrology.

[ref-9] Drbal K, Elster J, Komárek J (1992). Heavy metals in water, ice and biological material from Spitsbergen, Svalbard. Polar Research.

[ref-46] Franczak Ł, Kociuba W, Gajek G (2016). Runoff variability in the Scott River (SW Spitsbergen) in summer seasons 2012–2013 in comparison with the period 1986–2009. Quaestiones Geographicae.

[ref-10] Gong SL, Barrie LA (2005). Trends of heavy metal components in the Arctic aerosols and their relationship to the emissions in the Northern Hemisphere. Science of the Total Environment.

[ref-11] Grotti M, Soggia F, Ardini F, Bazzano A, Moroni B, Vivani R, Cappelletti D, Misic C (2017). Trace elements in surface sediments from Kongsfjorden, Svalbard: occurrence, sources and bioavailability. International Journal of Environmental Analytical Chemistry.

[ref-12] Harasimiuk M, Gajek G, Zagórski Piotr, Harasimiuk Marian, Rodzik J (2013). Tectonic and lithology. The Geographical Environment of W part of Wedel Jarlsberg Land (Spitsbergen, Svalbard).

[ref-13] Holt MS (2000). Sources of chemical contaminants and routes into the freshwater environment. Food and Chemical Toxicology.

[ref-14] Jensen J (1999). Fate and effects of linear alkylbenzene sulphonates (LAS) in the terrestrial environment. Science of the Total Environment.

[ref-15] Jóźwiak M (2007). The heavy metals in water of select Spitsbergen and Iceland glaciers. Landform Analysis.

[ref-16] Kociuba W (2017a). Determination of the bedload transport rate in a small proglacial High Arctic stream using direct, semi-continuous measurement. Geomorphology.

[ref-17] Kociuba W (2017b). Assessment of sediment sources throughout the proglacial area of a small Arctic catchment based on high-resolution digital elevation models. Geomorphology.

[ref-18] Kociuba W, Janicki G (2018). Effect of meteorological patterns on the intensity of streambank erosion in a proglacial gravel-bed river (Spitsbergen). Water.

[ref-19] Kociuba W, Janicki G, Dyer JL (2019). Contemporary changes of the channel pattern and braided gravel-bed floodplain under rapid small valley glacier recession (Scott River catchment, Spitsbergen). Geomorphology.

[ref-20] Kociuba W, Kubisz W, Zagórski P (2014). Use of terrestrial laser scanning (TLS) for monitoring and modelling of geomorphic processes and phenomena at a small and medium spatial scale in Polar environment (Scott River—Spitsbergen). Geomorphology.

[ref-21] Kosek K, Kozioł K, Luczkiewicz A, Jankowska K, Chmiel S, Polkowska Ż (2019). Environmental characteristics of a tundra river system in Svalbard—part 2: chemical stress factors. Science of the Total Environment.

[ref-22] Kozak K, Kozioł K, Luks B, Chmiel S, Ruman M, Marć M, Namieśnik J, Polkowska Ż (2015). The role of atmospheric precipitation in introducing contaminants to the surface waters of the Fuglebekken catchment, Spitsbergen. Polar Research.

[ref-23] Kozak K, Polkowska S, Luks B, Chmiel S, Ruman M, Lech D, Kozioł K, Tsakovski S, Simeonov V (2016). Arctic catchment as a sensitive indicator of the environmental changes: distribution and migration of metals (Svalbard). International Journal of Environmental Science and Technology.

[ref-24] Kozak K, Polkowska Z, Ruman M, Kozioł K, Namieśnik J (2013). Analytical studies on the environmental state of the Svalbard Archipelago provide a critical source of information about anthropogenic global impact. TrAC Trends in Analytical Chemistry.

[ref-25] Kozioł K, Uszczyk A, Pawlak F, Frankowski M, Polkowska Ż (2021). Seasonal and spatial differences in metal and metalloid concentrations in the snow cover of Hansbreen, Svalbard. Frontiers in Earth Science.

[ref-26] Lehmann S, Gajek G, Chmiel S, Polkowska Ż (2016). Do morphometric parameters and geological conditions determine chemistry of glacier surface ice? Spatial distribution of contaminants present in the surface ice of Spitsbergen glaciers (European Arctic). Environmental Science and Pollution Research.

[ref-27] Lehmann-Konera S, Franczak Ł, Kociuba W, Szumińska D, Chmiel S, Polkowska Ż (2018). Comparison of hydrochemistry and organic compound transport in two non-glaciated high Arctic catchments with a permafrost regime (Bellsund Fjord, Spitsbergen). Science of the Total Environment.

[ref-28] Lehmann-Konera S, Kociuba W, Chmiel S, Franczak Ł, Polkowska Ż (2019). Concentrations and loads of DOC, phenols and aldehydes in a proglacial arctic river in relation to hydro-meteorological conditions: a case study from the southern margin of the Bellsund Fjord—SW Spitsbergen. Catena.

[ref-29] Macdonald RW, Barrie LA, Bidleman TF, Diamond ML, Gregor DJ, Semkin RG, Strachan WMJ, Li YF, Wania F, Alaee M, Alexeeva LB, Backus SM, Bailey R, Bewers JM, Gobeil C, Halsall CJ, Harner T, Hoff JT, Jantunen LMM, Lockhart WL, Mackay D, Muir DCG, Pudykiewicz J, Reimer KJ, Smith JN, Stern GA, Schroeder WH, Wagemann R, Yunker MB (2000). Contaminants in the Canadian Arctic: 5 years of progress in understanding sources, occurrence and pathways. Science of the Total Environment.

[ref-30] Maenhaut W, Cornille P, Pacyna JM, Vitols V (1989). Trace element composition and origin of the atmospheric aerosol in the Norwegian arctic. Atmospheric Environment.

[ref-31] Michelutti N, Blais JM, Mallory ML, Brash J, Thienpont J, Kimpe LE, Douglas MSV, Smol JP (2010). Trophic position influences the efficacy of seabirds as metal biovectors. Proceedings of the National Academy of Sciences of the United States of America.

[ref-32] Nowak A, Hodson A (2013). Hydrological response of a High-Arctic catchment to changing climate over the past 35 years: a case study of Bayelva watershed, Svalbard. Polar Research.

[ref-33] Ottesen RT, Dallmann WK (2015). Geochemistry of superficial deposits. Geoscience Atlas of Svalbard.

[ref-34] Pacyna JM, Ottar B, Tomza U, Maenhaut W (1985). Long-range transport of trace elements to Ny Ålesund, Spitsbergen. Atmospheric Environment.

[ref-35] Pękala K (2004). Bellsund.

[ref-36] Ruman M, Kozak K, Lehmann S, Kozio K, Obecne Z, Komponentach N (2012). Pollutants present in different components of the svalbard archipelago environment—transport of pollutants throughout the Svalbard Archipelago. Ecological Chemistry and Engineering S.

[ref-37] Sagerup K, Savinov V, Savinova T, Kuklin V, Muir DCG, Gabrielsen GW (2009). Persistent organic pollutants, heavy metals and parasites in the glaucous gull (Larus hyperboreus) on Spitsbergen. Environmental Pollution.

[ref-38] Stachnik Ł, Yde JC, Nawrot A, Uzarowicz Ł, Łepkowska E, Kozak K (2019). Aluminium in glacial meltwater demonstrates an association with nutrient export (Werenskiöldbreen, Svalbard). Hydrological Processes.

[ref-39] Stanisz A (1998). Przystępny kurs statystyki w oparciu o program STATISTICA PL na przykładach z medycyny [STATISTICA PL-based accessible course on statistics on medical examples].

[ref-40] Szumińska D, Szopińska M, Lehmann-Konera S, Franczak Ł, Kociuba W, Chmiel S, Kalinowski P, Polkowska Ż (2018). Water chemistry of tundra lakes in the periglacial zone of the Bellsund Fiord (Svalbard) in the summer of 2013. Science of the Total Environment.

[ref-41] Vaughan DG, Comiso JC, Allison I, Carrasco J, Kaser G, Kwok R, Mote P, Murray T, Paul F, Ren J, Rignot E, Solomina O, Steffen K, Zhang T, Stocker TF, Qin D, Plattner GK, Tignor M, Allen SK, Boschung J, Nauels A, Xia Y, Bex V, Midgley PM (2013). Observations: cryosphere. Climate Change 2013: The Physical Science Basis. Contribution of Working Group I to the Fifth Assessment Report of the Intergovernmental Panel on Climate Change.

[ref-42] Wenzel C, Gabrielsen GW (1995). Trace element accumulation in three seabird species from Hornøya, Norway. Archives of Environmental Contamination and Toxicology.

[ref-43] Zaborska A, Beszczyńska-Möller A, Włodarska-Kowalczuk M (2017). History of heavy metal accumulation in the Svalbard area: distribution, origin and transport pathways. Environmental Pollution.

[ref-44] Zhang R, John SG, Zhang J, Ren J, Wu Y, Zhu Z, Liu S, Zhu X, Marsay CM, Wenger F (2015). Transport and reaction of iron and iron stable isotopes in glacial meltwaters on Svalbard near Kongsfjorden: from rivers to estuary to ocean. Earth and Planetary Science Letters.

[ref-45] Ziółek M, Bartmiński P, Stach A (2017). The influence of seabirds on the concentration of selected heavy metals in organic soil on the Bellsund Coast, Western Spitsbergen. Arctic, Antarctic, and Alpine Research.

