# Supplementary material for: Effects of biotransport and hydro-meteorological conditions on transport of trace elements in the Scott River (Bellsund, Spitsbergen)
Source: PeerJ. 2021 Jun 28;9:e11477. doi: 10.7717/peerj.11477 (PMC8247700; doi:10.7717/peerj.11477)
Supplement: Supplemental Information 1 [file peerj-09-11477-s001.docx]

**Supplementary file 1**

In the Scott River, the measurement profile was located 200 m above its mouth to the Bellsund Fjord. Water stages were recorded 144 times per day with a CTD-Diver meter (Schlumberger Water Services), with a measurement accuracy of ±0.5cm. The flow velocity was measured by a HEGA II current meter and an OTT ADC ultrasonic device, with a range of flow velocity measurements of 0.02– 3.00 m s^−1^ and 0.2–2.4 m s^−1^ (and an accuracy of ±0.25 cm s^−1^) respectively (Franczak et al., 2016). In the case of measured water discharge (Q) in the Scott River gorge, we decided to treat these measurements as corresponding to the amount of transported water at the mouth of the river due to the very short distance (200 m) between the gorge and river mouth (Lehmann-Konera et al., 2019).

Meteorological measurements covering the registration of air temperature (T) were performed every 10 minutes by means of an automatic meteorological station (Campbell Scientific CR10 datalogger). For the measurements of atmospheric precipitation (P) a Hellman rain gauge, with 200 cm^2^ of inlet ring, was used. Both the meteorological station and the precipitation sampler were placed approximately 1 km from the hydrometric station, within the elevated marine terrace Calypsostranda, at an altitude of 23 m a.s.l.(Lehmann-Konera et al., 2019).

**References**

Franczak, L., Kociuba, W., Gajek, G., 2016. Runoff Variability in the Scott River (SW Spitsbergen) in Summer Seasons 2012-2013 in Comparison with the Period 1986-2009. Quaest. Geogr. 35, 39–50. https://doi.org/10.1515/quageo-2016-0025

Lehmann-Konera, S., Kociuba, W., Chmiel, S., Franczak, Ł., Polkowska, Ż., 2019. Concentrations and loads of DOC, phenols and aldehydes in a proglacial arctic river in relation to hydro-meteorological conditions. A case study from the southern margin of the Bellsund Fjord – SW Spitsbergen. Catena. https://doi.org/10.1016/j.catena.2018.10.049
