# Supplementary material for: Effects of biotransport and hydro-meteorological conditions on transport of trace elements in the Scott River (Bellsund, Spitsbergen)
Source: PeerJ. 2021 Jun 28;9:e11477. doi: 10.7717/peerj.11477 (PMC8247700; doi:10.7717/peerj.11477)
Supplement: Supplemental Information 3 — n.d.- not determined, (N)-number of samples with results >LOD used for loads calculation, * data after Lehmann-Konera et al., 2019. [file peerj-09-11477-s003.docx]

**Supplementary file 3.** **Data of minimum and maximum values of determined trace elements in freshwater samples collected from the studied catchment.** n.d.- not determined, (N)-number of samples with results >LOD used for loads calculation, * data after Lehmann-Konera et al., 2019.

| **Determined analytes** | **Concentration ranges [µg/L]** | | **Load ranges [µg/s] (N)** | |
| --- | --- | --- | --- | --- |
|  | **Gorge** | **Mouth** | **Gorge** | **Mouth** |
| Ag | <LOD-0.012 | <LOD | 13.0 **(1)** | n.d. **(0)** |
| Al | 0.163-5.71 | 0.124-5.71 | 53.6-10 537 **(42)** | 73.8-7 113 **(42)** |
| As | <LOD | <LOD-0.121 | n.d. **(0)** | 6.98-72.7 **(38)** |
| Ba | 0.290-2.75 | 0.524-2.49 | 425-3 798 **(42)** | 349-3 883 **(42)** |
| Be | <LOD-0.017 | <LOD-0.012 | 11.6-18.4 **(3)** | 12.3 **(1)** |
| Cd | <LOD-0.091 | <LOD | 4.40-81.9 **(16)** | n.d. **(0)** |
| Co | 0.011-0.057 | 0.010-0.060 | 7.98-71.7 **(42)** | 6.41-74.7 **(42)** |
| Cr | <LOD-0.047 | <LOD-0.100 | 5.72-50.8 **(10)** | 8.26-39.4 **(14)** |
| Cs | <LOD | <LOD | n.d. **(0)** | n.d. **(0)** |
| Cu | <LOD-0.425 | 0.014-0.101 | 5.20-454 **(33)** | 10.2-80.8 **(42)** |
| Ga | 0.010-0.029 | 0.011-0.027 | 5.13-48.3 **(41)** | 4.40-40.8 **(42)** |
| Hg | <LOD | <LOD | n.d. **(0)** | n.d. **(0)** |
| La | <LOD | <LOD | n.d. (**0)** | n.d. **(0)** |
| Li | 0.253-0.573 | 0.193-0.675 | 149-671 **(42)** | 166-793 **(42)** |
| Mn | 0.017-17.7 | 0.082-12.0 | 18.5-9 749 **(42)** | 48.2-13 888 **(42)** |
| Ni | <LOD-0.406 | 0.011-0.334 | 4.00-365**(35)** | 5.20-302 **(42)** |
| Pb | <LOD-0.020 | <LOD-0.052 | 3.14-17.5 **(9)** | 3.77-76.3 **(8)** |
| Rb | 0.048-0.244 | 0.053-0.144 | 22.5-260 **(42)** | 23.7-202 **(42)** |
| Se | 0.012-0.199 | 0.058-0.190 | 10.6-300 **(42)** | 35.9-296 **(42)** |
| Sr | 10.3-40.6 | 11.6-36.2 | 7 407-55 956 **(42)** | 5 893-50 058 **(42)** |
| Th | <LOD-0.016 | <LOD-0.011 | 17.3 **(1)** | 11.9 **(1)** |
| Tl | <LOD-0.010 | <LOD-0.013 | 8.53 **(1)** | 10.9-11.3 **(2)** |
| U | 0.027-0.248 | 0.022-0.134 | 20.0-212 **(42)** | 18.7-226 **(42)** |
| V | <LOD-0.067 | <LOD-0.030 | 3.99-57.2 **(28)** | 5.26-65.8 **(16)** |
| Zn | <LOD-1.88 | <LOD-0.736 | 61.9-1 503 **(32)** | 58.4-906 **(17)** |
| **Ʃmetals** | 12.6-54.9 | 15.3-41.9 | 9 459-62 995 | 7 195-57 817 |
| **Chemical parameters*** | |  |  |  |
| pH [-] | 7.47-8.79 | 7.41-8.81 | n.d. | n.d. |
| DOC [mg/L] | <LOD-0.213 | <LOD-0.395 | 9.41-279 **(24)** | 16.4-579 **(30)** |
| **Hydrological parameter for the gorge and mouth sections of the Scott River *** | | | | |
| Q [m^3^ s^-1^] | 0.285-2.19 | | | |
